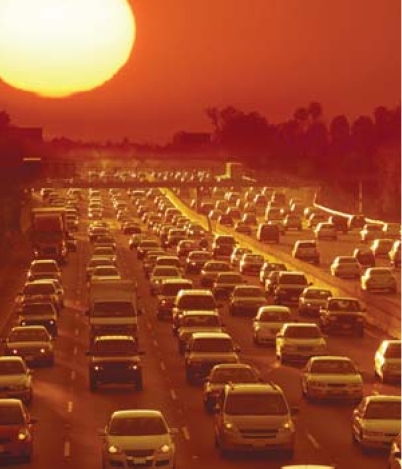# Innovative Technologies: Transportation Fuels on the Table

**DOI:** 10.1289/ehp.116-a67a

**Published:** 2008-02

**Authors:** Kellyn S. Betts

On 29–30 November 2007, just days before the United Nations Climate Change Conference began in Bali and Al Gore and Rajendra Pachauri received the 2007 Nobel Peace Prize for their work on climate change, the U.S. Institute of Medicine (IOM) held a workshop to highlight gaps in scientific understanding about the environmental and human health effects of transportation fuels. The transportation sector’s use of fuel is expected to grow more quickly than demand for energy in any other sector between 2005 and 2030, Scott Nauman, manager of economy and energy in the ExxonMobil Corporate Planning Department, told conference attendees. Therefore, new transportation fuels are key players among the innovative technologies being investigated and developed to help countries throughout the world cut greenhouse gas emissions.

As worldwide transportation fuel use rises, the emissions and carbon dioxide emitted by burning these fuels will also rise, pointed out Samuel Wilson, acting director of the NIEHS. “We want to add ‘minimizing the human health impact’ . . . to the criteria for developing alternative fuels,” he said.

According to Michael Wang, a vehicle and fuel systems analyst at the U.S. Department of Energy Argonne National Laboratory, biofuels are considered the most promising alternative for reducing petroleum use and decreasing greenhouse gas emissions from transportation. Electricity is also expected to grow as a source for plug-in hybrids, as is hydrogen. The idea of producing transportation fuels from coals “has generated some interest lately because they can be produced domestically,” Wang says, but their carbon emissions are “pretty high.”

The main biofuel currently being developed for transportation in the United States is ethanol from corn via distillation, and its production is increasing rapidly. According to the Energy Information Administration, the percentage of the U.S. gasoline pool represented by ethanol rose from 1.27% in 2000 to 2.85% in 2005. However, John Regalbuto, director of the National Science Foundation’s Catalysis and Biocatalysis Program, predicts that hydrocarbon-based “green gasoline,” “green diesel,” and “green jet fuel” will represent a significant fraction of new biofuels by 2022. When produced via catalysis from materials such as switch-grass and waste wood products, the performance of these renewable biofuels are “essentially the same” as their conventional counterparts, he says. However, their manufacture involves intermediates such as hydroxymethylfurfural and ionic liquid solvents that require further study in terms of health effects.

Additives also affect the emissions of transportation fuels, as well as these fuels’ impact on the environment, stressed Serap Erdal, an assistant professor at the University of Illinois–Chicago School of Public Health. Uncertainty over which additives will be added to new fuels and how the complex mixtures will behave in the environment makes it very hard to predict potential health effects, agreed the experts amassed at the IOM meeting. In the United States, for example, ethanol has only recently been used as an additive to make gasoline burn more efficiently. But in Brazil, where ethanol has been used as a fuel for nearly 30 years, it has become clear that, compared with gasoline, the sugarcane-derived fuel produces much higher emissions of formaldehyde, a known human carcinogen, and acetylaldehyde, a suspected human carcinogen, said Paolo Saldiva, a professor of medicine in the University of São Paolo’s Department of Pathology. However, he said that the health effects of exposure to these compounds via air emissions are unclear.

What is indisputable is that key decisions leading to the development of new fuels will be made before the requisite health effects data are available, so “we need to get going as soon as possible . . . to collect baseline data,” said Dan Greenbaum, president of the Health Effects Institute, which published a report summarizing the health effects of exposure to 21 mobile source air toxics in November 2007. Added Erdal, “There is a critical need to institute public health surveillance so that we are already collecting data and are able to detect changes as they take place.”

## Figures and Tables

**Figure:**